# COVID-19 Infection Clinical Profile, Management, Outcome, and Antibody Response in Kidney Transplant Recipients: A Single Centre Experience

**DOI:** 10.1155/2021/3129411

**Published:** 2021-10-03

**Authors:** Sanjiv Jasuja, Gaurav Sagar, Anupam Bahl, Shalini Verma

**Affiliations:** ^1^Deaprtment of Nephrology, Indraprastha Apollo Hospitals, New Delhi, India; ^2^AVATAR Foundation, Department of Clinical Research, New Delhi, India

## Abstract

**Introduction:**

Experience of COVID-19 in kidney transplant recipients (KTRs) with clinical presentation, management, factors influencing mortality, and antibody response is limited. *Material and Methods*. A retrospective data of COVID-19 in KTRs was collected and analyzed. The mortality rate, risk factors, and antibody response were primary objectives, while the clinical presentation, laboratory indicators, and pharmacological management were secondary objectives.

**Results:**

The 67 KTRs with polymerase chain reaction (PCR) confirmed COVID-19 infection reported between 1 May 2020 and 31 December 2020; 61.2% of patients were hospitalized; and 20.9% needed ventilation. The overall mortality was 26.9%, while blood group A had 50% mortality. The treatment options and used were steroids (100%), convalescent plasma (32.8%), ivermectin (58.2%), doxycycline (55.2%), remdesivir (34.3%), tocilizumab (10.4%), antibiotics (61.2%), anti-fungals (26.9%), low molecular weight heparin (45.3%), and oral anti-coagulants (26.9%). Anti-nucleosides (mycophenolate or azathioprine) were discontinued in 76.1% and calcineurin inhibitors (CNI) in 26.9%. Significant mortality (*p* < 0.001) was observed in patients presenting with SpO_2_ <94 needing ICU care, ventilation, dialysis/acute kidney injury (AKI), and empirical therapies like convalescent plasma and remdesivir. The age of survivors versus nonsurvivors was not significantly different (*p*=0.02). The positive blood culture, low serum albumin, high TLC, high blood urea, interleukin-6, and CT severity score ≥15 were statistically significant in nonsurvivors. Overall mortality, mortality of hospitalized patients, and mortality of ventilated patients was 27%, 44%, and 100%, respectively. The median value of SARS-CoV-2 (COVID-19) IgG antibody was 68.60 (IQR, 28.5–94.25) AU/ml in more than 90% of survivors.

**Conclusion:**

KTRs with COVID-19, needing ICU care, dialysis and ventilation support had poor outcomes. Recovered patients mounted adequate antibody response.

## 1. Introduction

Infection with severe acute respiratory syndrome coronavirus 2 (SARS-CoV-2) mainly affects the respiratory tract but does not spare other organs, brain and kidneys being the major targets. Clinical management of COVID-19 infection in kidney transplant recipients (KTRs) calls for early diagnosis and aggressive treatment strategy for a favourable outcome in otherwise reported high mortality group. Kidney injury is well documented with COVID-19, and KTRs are more vulnerable for rapid renal function deterioration. The immunocompromised state, post-transplant duration, age, comorbidities, treatment in a nontransplant centre, and frailty add to the risk factors for poor outcomes [1, 2].

The COVID-19 may prove fatal to KTRs with 28% mortality (range 16%–30%) [1, 3, 4] compared to 1%–5% in the general population [5]. The lymphopenia and hypoxemia (SpO_2_ < 94%) at presentation are associated with high mortality in the hospitalized KTRs [2]. Additionally, the KTRs have a high viral load for a longer duration. It is important to monitor these patients until the viral load is negligible by polymerase chain reaction (PCR) to prevent disease spread in the community.

The exact role of immunosuppression on the progression of COVID-19 is unknown, but it cannot be denied due to associated high mortality. A few recently published case series of COVID-19 in KTRs propose minimizing immunosuppression while continuing steroid therapy [6]. The judicial dose reduction or discontinuation of immunosuppressants needs close supervision.

As experience with COVID-19 in KTRs is limited, there is a need for collective data. We are sharing our experience of KTRs with COVID-19 with a focus on the modifiable and nonmodifiable risk factors, laboratory investigation including inflammatory markers, morbidity profile, response to pharmacological intervention, antibody response, and mortality. The study observations may help in better treatment approach and management of COVID-19 in this population.

## 2. Methods

This retrospective analysis on COVID-19 disease in 67 kidney transplant recipients (KTRs) treated between 1 May 2020 and 31 December 2020 was carried out in the nephrology unit of a tertiary care hospital in New Delhi, India. The aim of this study was to describe the clinical symptoms, risk factors, laboratory profile, disease management, mortality, and antibody response to COVID-19 in KTRs. All the patients were followed up for a minimum of 90 days excluding deaths. Assessing the mortality rate, correlating with risk factors, and IgG antibody response to COVID-19 were the primary objectives; describing the spectrum of clinical presentation, laboratory investigations, and pharmacological management were the secondary objectives.

Data were obtained from the medical records of the hospitals or patient's follow-up submissions. The KTRs with a history of fever, cough, and PCR positive COVID-19 were investigated and managed by a designated COVID-19 treating team in consultation with the treating nephrologist. The diagnosis was based on the guidelines of the World Health Organization [7, 8]. The data on demography, symptoms, laboratory investigations, treatment received, therapeutic outcomes (mortality and recovery), and IgG antibody response were collected. Patients were categorized into obese, overweight, normal, and underweight with body mass index (BMI) criterion calculated as per consensus group recommendation for BMI in Asian population [9].

In all cases at presentation, clinical symptoms and disease severity were recorded as per the Chinese Centre of Disease Control (CDC) [10] criterion by the treating team. The mild disease include nonpneumonia or mild pneumonia (mild symptoms without dyspnoea, respiratory rate <30/min, blood oxygen saturation (SpO_2_) >93%, and PaO_2_/FiO_2_ ratio ≥300 mmHg). The severe disease had dyspnoea, respiratory rate ≥30/min, SpO_2_ ≤93%, PaO_2_/FiO_2_ ratio <300 mmHg, and/or lung infiltrates >50% within 24 to 48 h (in our study, PaO_2_/FiO_2_ ratio was not done in domiciliary patients, while chest X-ray at admission was not done in any patient). The definition of critical disease included adult respiratory distress syndrome (ARDS) or respiratory failure, septic shock, and/or multiple organ dysfunction (MOD) or failure (MOF).

**Figure 1:**
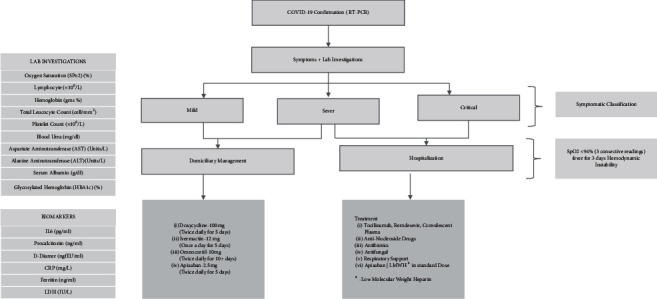
Schematic representation of the study in kidney transplant recipients with COVID-19.

In COVID-19, PCR-confirmed KTRs with mild disease and/or having low-grade fever, cough, and/or myalgia were followed up telephonically. These patients were advised to adhere to the domiciliary quarantine guidelines including self-isolation, monitoring body temperature, vitals, oxygen saturation, periodic investigations, and drug treatment as per annexure 1. Patients were advised to consult with a transplant physician or designated COVID-19 physician if there was any worsening in their symptoms and also to discuss and share their laboratory reports at periodic intervals. Schematic representation of the study activities is provided in Figure 1.

Approximately 10% of completely asymptomatic domiciliary patients were noncompliant for one or all of the prescribed drugs or investigation protocol, but their symptoms and the clinical course was recorded and included in this study. The sustained hypoxia with three consecutive peripheral oxygen saturation readings below 94% while on room air 60 minutes apart, continuous fever for 3 days, or any haemodynamic instability were the indications for hospitalization.

The demography and symptoms for domiciliary patients were recorded telephonically on first clinical reporting. While for hospitalized patients, these were sourced from triage notes. All patients were evaluated as per unit protocol, summarized in Figure 1. When more than one laboratory parameter values were available, the mean of all available values was used for study corelations.

Post-hospitalization patients were assessed initially at triage, then on regular basis for oxygen requirement in wards. Patients needing >10 litre/minute flow of oxygen, by mask or nasal prongs, to maintain oxygen saturation or having other parameters of critical disease as mentioned above were shifted to intensive care unit (ICU) for further treatment. Unless contraindicated, these patients received remdesivir, tocilizumab, and convalescent plasma (two doses). Patients were treated with appropriate anti-microbials depending on clinical condition, white cell counts, blood and urine cultures, infection biomarkers (CRP, procalcitonin trends, and ferritin), and/or radiological imaging. All patients received steroids and oral anti-coagulation or low molecular weight heparin (LMWH) or conventional heparin, unless contraindicated.

The need for respiratory support was proportionate to hypoxia of individual patient, which varied from high flow oxygenation in ward to prone ventilation in intensive care unit (ICU). AKI was defined using the KDIGO-2012 [11] criterion with baseline serum creatinine. The CT scanning was done when patients despite ongoing treatment worsened their oxygen saturation and CT findings were quantified on the basis of CT severity score index [12]. The PCR test was repeated at 15 days frequency till negative on two consecutive days. Post-recovery all patients were subjected to SARS-CoV-2 (COVID-19) IgG quantitative antibody assessment after the availability of test in India as a surrogate for immune response. At the time of this data compilation, anti-spike SARS-CoV-2 IgG antibody test was not routinely available. These tests were conducted 3 weeks to 30 weeks post-onset of COVID-19 infection.

### 2.1. Statistical Analysis

Pooled data was captured on Microsoft Excel and imported to SPSS statistical software version 16.0 (SPSS Inc., Chicago) for analysis, and “boot” package available in R-software version 3.6.1 was applied for determining the median difference confidence interval. Descriptive statistics of continuous variables were summarized as mean ± standard deviation (SD) and median (interquartile range). The qualitative variables were reported with numbers and percentages. The unpaired Student's *t*-test was applied to compare the mean between survivor and nonsurvivor groups for normally distributed variables; for inflammatory markers and some biomarkers, the nonparametric Mann–Whitney *U* test was applied due to skewed distribution. The chi-square and Fisher's exact were applied to find the association between mortality and qualitative variables. The mean difference and 95% confidence interval (CI) were reported for normally distributed variables and median difference and its 95% confidence interval for skewed variables using the bootstrapping method with 10,000 bootstrapping samples. The binary logistic regression was performed to determine the odds ratio and its 95% CI. To find the optimal cut-off and discriminate power of biomarkers, receiver operating characteristic (ROC) curve was applied. The Youden index criteria were used for determining the optimal cut-off for the respective biomarker. Multivariable logistic regression was not performed to find the independent risk factors for nonsurvivor due to the small number of cases and missing values of biomarkers, and also some of the variables had zero count. To understand the time to response of IgG antibodies and sustenance, the various relationships between the antibody values and duration of antibody development from the onset of COVID-19 disease were fitted using curve fitting analysis. The *p*-value less than 0.001 was considered as significant; Bonferroni correction was applied keeping a small sample size in consideration and multiple variable testing. We excluded patients from antibody analysis who had received convalescent plasma during treatment.

## 3. Results

There were 67 kidney transplant recipients (KTRs): males 50 and females 17 (male:female – 1:0.34) with PCR-confirmed COVID-19. The mean (SD) age of patients was 51.34 (13.0) years, range 18–79 years. Mean (SD) BMI was 21 (4.21) kg/m^2^, while 18 (26.9%) and 15 (22.4%) patients were underweight and overweight, respectively (Tables 1 and 2 ).

Lymphopenia was observed in 16 (23.8%) patients. The average post-transplant period (PTP) was 297.25 weeks (see Table 3 for laboratory investigations).

Mean (SD) weight and height were 70.34 (14.99) kg and 1.67 (0.08) meters, respectively; there was a no statistically significant gender difference in weight: males 71.20 (13.74) kg and females 57.29 (18.59) kg (*p*=0.360), but it was significant in height: males 1.70 (0.07) meter and females 1.59 (0.05 meter; *p* < 0.001), which may not be clinically relevant. The blood group distribution among the infected patients were 32.8% (*n* = 22), 28.4% (*n* = 19), 32.8% (*n* = 22), and 6% (*n* = 4) for groups O, A, B, and AB, respectively (Table 1).

### 3.1. Clinical Presentation

Fever (*n* = 59, 88.1%) and cough (*n* = 47, 70.1%) were the major symptoms reported; breathing difficulty (*n* = 26, 38.8%) and body ache (*n* = 25, 37.3%) were other main complaints; four patients (6%) were detected incidentally with COVID-19. Hypertension (HTN – *n* = 61, 91%) and type 2 diabetes mellitus (DM – *n* = 34, 50.7%) were the most common comorbidities (Table 1). Concurrent cytomegalovirus (CMV) activation (*n* = 03, 4.5%), blood culture positivity (*n* = 05, 7.5%), and urine culture positivity (*n* = 04, 6%) were observed along with COVID-19.

### 3.2. Management

Forty-one (61.2%) patients required hospitalization; only 14 (20.9%) needed ventilator support; and ∼50% were managed without any respiratory support. Steroids (*n* = 67, 100%) were the core treatment. Ivermectin (*n* = 39, 58.2%), doxycycline (*n* = 37, 55.2%), and remdesivir (*n* = 23, 34.3%) were other coadministered drugs. Convalescent plasma was given to 22 (32.8%) hospitalized patients. Antibiotics and anti-fungals were used in 61.2% (*n* = 41) and 26.9% (*n* = 18), respectively, either empirically in terminally sicker patients or based on suggestive imaging or on specific organism grown on culture. Low molecular weight heparin (LMWH; *n* = 31, 45.3%), oral anti-coagulants (*n* = 18, 26.9%), and anti-platelet agents (*n* = 02, 3.0%) were prescribed for thromboprophylaxis. Anti-nucleoside drugs were discontinued in 51 patients (76.1%); dose reduced in three patients (4.5%). Tacrolimus or cyclosporine was continued in same or reduced dose in 49 patients (73.1%) and stopped in 18 patients (26.9%). Thirty-eight percent KTRs (24/63) had AKI; out of these 54.2% (13/24) needed dialysis, and 66.7% (16/24) died. Computerized tomography (CT) scanning was done in only 30 patients, and out of these, 50% patients (*n* = 15) had CT severity score ≥15 (Table 4).

### 3.3. Outcome

Forty Patients (59.7%) recovered completely; nine (13.4%) had mild-to-moderate respiratory sequelae after recovery. Eighteen (27%) patients succumbed to COVID-19 either due to active disease (15, 22.5%) or due to post-COVID-19 sequelae (3, 4.5%). The significant mortality (*p* < 0.001) was observed in patients on ventilator support (14/14, 100%) and received convalescent plasma (*n* = 15/18, 83.3%) and remdesivir (*n* = 14/18, 77.8%).

Positive blood culture, anti-fungal treatment, higher blood urea, lower serum albumin, and low oxygen saturation at presentation (SpO_2_ < 94) were significant in nonsurvivors (*p* < 0.001; Table 2). The 50% of nonsurvivors had blood group A (9/18).

**Figure 2:**
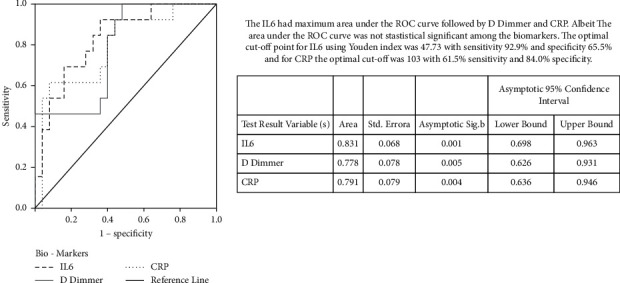
ROC curve for diomarkers IL-6, D-dimer, and CRP in kidney transplant recipients with COVID-19.

The inflammatory markers procalcitonin, CRP, D-dimer, ferritin, and LDH were not statistically significant, while IL-6 levels were significantly higher in nonsurvivors than survivors. The receiver operating characteristics (ROC) curve analysis revealed among the significant inflammatory biomarkers, and IL-6 had maximum discriminatory power (Figure 2). The average duration of active COVID-19 among survivors was 29.43 days.

### 3.4. Antibody Response

The SARS-CoV-2 (COVID-19) IgG antibodies by chemiluminescence immunoassay (CLIA; LIAISON SARS-CoV-2 S1/S2, DiaSorin, Italy) were measured in 49 patients over 6 months after recovery, while 8 patients had received convalescent plasma during treatment were excluded from final analysis. The median value obtained was 68.6 (interquartile range (IQR), 28.5–94.25) AU/ml. The 9.8% patients (4/41) had antibody response below 12 AU/ml, indicating the absence of SARS-CoV-2 IgG antibodies. The curve fitting analysis found no significant relationship between the antibody values and duration of antibody development from the onset of COVID-19.

## 4. Discussion

Kidney transplant recipients with severe COVID-19 require hospitalization due to rapid disease progression, while the need for intensive care and/or haemodialysis increases the risk of mortality. The disease-associated cytokine release syndrome leads to multiorgan dysfunction including acute kidney injury (AKI) in these patients [13]. The laboratory abnormalities including lymphopenia; elevated acute biochemical markers, that is, C-reactive protein; procalcitonin; interleukin-6; *D* dimer; and radiological findings of ground glassing; pneumonia; and fibrosis are associated with poor prognosis [14]. The nonmodifiable clinical spectrum associated with poor outcomes includes obesity, pre-existing respiratory disease, hypertension, male gender, age >60 years, hypertension, diabetes mellitus, tobacco smoking, pre-existing cardiac diseases, and the first year after transplantation [3, 5, 15]. Elhadedy et al. reported, in their series, that more than 90% KTRs required hospitalization, and dyspnoea was the chief complaint in 80% needing ICU service [15].

The mean age of our study population was >50 years with male predominance (75%) and the median (25^th^ to 75^th^ percentile) of post-transplant duration was 260.0 weeks (IQR, 133.0–390.0 weeks). The predominant comorbidities associated were type 2 diabetes mellitus (51%), hypertension (91%), vascular disease (26%), and chronic allograft dysfunction (31%). Four asymptomatic cases were detected incidentally while going through COVID-19 screening as a mandatory prerequisite for other ailment hospitalization during the pandemic.

The clinical spectrum of COVID-19 disease in KTRs reported in literature ranged from being asymptomatic to presenting with fever, cough, dyspnoea [2, 15–18], diarrhoea [5], myalgia, chills, fatigue [17], and need for hospitalization (80%) [16]. The semiology of symptoms in our patients was in line with the literature with fever (88.1%), cough (70.1%), breathing difficulty (38.3%), and body aches (37.3%). We observed blood groups O and B (32.8% each) were more affected. The concurrent cytomegalovirus (CMV) activation or infection was observed in 5% of patients.

The reported incidence of AKI and abnormal renal parameters in COVID-19 patients in the general population is 3%–9% [19–22], While the risk of development of AKI in KTRs with COVID-19 is very high (42%) [23]. We observed in our patients that approximately 40% of KTRs with COVID-19 developed AKI, more than 50% of them needed dialysis support, and two-third of the patients succumbed in this group.

The literature has shown significant corelation between various laboratory parameters, clinical spectrum, and outcome. The degree of leucopenia has been associated with disease severity, acute respiratory syndrome (ARDS) [24], and fatal outcome [25, 26]. Similarly, high CRP levels corelates with the severity of COVID-19 [20], ARDS [24], myocardial damage, and mortality [27]. The higher ferritin levels have been associated with ARDS [25] and death [28]. The IL-6, a novel biomarker for COVID-19 severity, has been correlated with mortality in various studies [21, 24]. In our study, low oxygen saturation at presentation, high blood urea, lower serum albumin, the higher median value of inflammatory marker-IL-6, and high total leucocyte count (TLC) were statistically significant for nonsurvivors (Table 2).

The chest X-ray or computerized tomography (CT scan) assesses the extent of viral pneumonia indicating COVID-19 severity; these changes corelates well with hypoxia and outcome [6]. In our study, a CT scan was done in 30 patients; a severity score of ≥15 was associated with 60% mortality.

In published data, mortality of KTRs due to COVID-19 is significantly high (KTRs, 24% vs. 1% in the general population) [23], particularly those on dialysis for a longer time prior to KT [29] and needing hospitalization [5, 15, 30]. Around 61.2% of our patients required hospitalization; mortality in this group was 44%, while it was 27% overall. Our overall mortality was higher compared to large multicentric pooled data and publications from public sector hospitals from India (27% vs. 11.6% and 9.5%) [31]. This difference may be due to longer follow-up duration and deaths due to COVID-19 lung sequel. In our study and both of these studies, mortality for ventilated patients was 100%.

In the absence of a definite therapy or guidelines, the modification of immunosuppressants [5] and continuation of steroids along with other COVID-19 management strategies are recommended for KTRs [32]. The other COVID-19 treatment used in KTRs includes anti-virals, hydroxychloroquine, macrolides, remdesivir, tocilizumab, and convalescents plasma as per unit protocol or as per the experience of treating physicians [33]. However, there are equivocal results for COVID-19 outcomes with these drugs in the general population [34–41] and KTRs [5, 12]. There is a thin line of separation between controlling COVID-19 with these empirical therapies or lowering immunosuppression and their impact on the kidney allograft functioning, directly or by inciting rejection. Hence, one has to decide very diligently on the choice of pharmacotherapy or immunosuppressant dose modification. According to Massachusetts General Hospital COVID-19 treatment guidelines, immunosuppression modification includes a reduction in the dose of calcineurin inhibitors by 50% and stopping anti-metabolites [42]. Kataria et al. and other authors have outlined the various treatment options including administration of hydroxychloroquine, IL-6 antagonist (tocilizumab), and RNA polymerase inhibitor (remdesivir) along with immunosuppression dose adjustment or withdrawal [3, 16, 17]. Among specific anti-viral therapy, remdesivir has shown good therapeutic result in some studies [43–45]. Convalescent plasma therapy has not been very promising, awaiting more evidence to prove its efficacy, until then its role is limited to severe COVID-19.

In our study, anti-nucleoside drugs (MMF and azathioprine) were stopped or reduced by more than 80%, while CNI was stopped by 27%. The other therapies used in our cohort included steroids (100%), ivermectin (58.2%), doxycycline (55.2%), remdesivir (34.3%), tocilizumab (10.4%), and convalescent plasma (32.8%) as per the unit protocol. The outcome with convalescent plasma was not very promising in our experience as mortality in this group was 63%. The anti-fungal treatment was given based on culture positivity or radiological suggestion or empirical grounds in 30% of patients. The patients receiving anti-fungal treatment were significantly high in the nonsurvivor group indicating the adverse prognostic impact of these infections.

The four reported outcomes of COVID-19 in literature in KTRs are: (i) uneventful asymptomatic disease, (ii) complete disease recovery, (iii) recovery with sequelae, and (iv) death (due to active disease or sequelae) [43]. In our study 73% recovered; 22.5% died due to active COVID-19; and 4.5% died due to its sequelae.

Significant mortality was observed in critical patients in our study, on the ventilator, treated with convalescent plasma, dialysis, and remdesivir. As described in methods, these modalities were used in patients not maintaining oxygen saturation or haemodynamic with standard care. Our data do not support the use of remdesivir and plasma therapy in the treatment of COVID-19 as reported in the literature [46].

We observed high mortality associated with blood group A in KTRs. The possible reasons for this observation are not clear. In a meta-analysis of community COVID-19 patients by Nanyang *L* et al., the individuals with blood group A were more prone to develop the disease with unfavourable outcomes [47]. Interestingly, studies have shown anti-A antibodies inhibit binding of glycosylated SARS-CoV S protein-expressing cells to angiotensin-converting enzyme 2 (ACE2) on the cell surface and hence provide protection by block the interaction between the virus and its receptors; these antibodies missing in blood group A patients. Additionally, the link between blood group A and higher susceptibility of thromboembolism, diabetes, hypertension, recurrent urinary tract infections from *Escherichia coli*, and gastric ulcers with *Helicobacter pylori* can also explain high mortality of severe COVID-19 in blood group A [48].

There is limited data available for short- and long-term antibody response to COVID-19 in immunocompromised KTRs [30, 49]. As per published literature, the duration of up to six months post infection is important to observe the trend of antibodies [50]. An antibody mounting response was noted for more than 90% of survivors in our study.

The main limitation of our study was that multivariate analyses could not be performed due to the small number of participants and hence the factors associated with mortality in the KTR population could not be assessed; however, Bonferroni correction was applied to keep a small sample size in consideration. We suggest conducting a statistically powered study to understand this antibody trend in KTRs and overall outcomes. The other limitation is only KTR were included in the study, so findings are not applicable to other organ transplants. The level of protection and duration of persistence of these antibodies and the efficacy of the vaccine is still not clear; these queries will settle over a period of time with a better understanding of COVID-19. With the vaccine for COVID-19 being available, KT recipients must be prioritized for vaccination, which may bring down the infection rate, associated complications, disease severity, and mortality.

## 5. Conclusion

Hospitalization, AKI, high CT score, activation of opportunistic infections, need for ventilation and dialysis, treatment with convalescent plasma, and remdesivir were associated with higher mortality due to COVID-19 in KTRs. Out of biomarkers, IL-6 correlates significantly with mortality. Half of the patients in the mortality group had blood group A. The IgG antibody response was noted post-COVID-19 in more than 90% of patients. Immunosuppression should be tailored by either discontinuation or dose reduction of anti-nucleosides and CNI, while high dose steroids can compensate immunosuppression reduction with the contribution to the outcome [51, 52].

## Figures and Tables

**Table 1:** Characteristics, comorbidities, and symptoms of kidney transplant recipients with COVID-19.

Category	Parameter	Total (*N* = 67), *n* (%)	Survivor *N* = 49 (% distribution in the group)	Nonsurvivors *N* = 18 (% distribution in the group)	Odds ratio (95% CI)^*∗*^	*P*-value^*∗*^
Gender	Male	50 (74.6)	37 (75.5)	13 (72.2)	0.84 (0.25–2.86)	0.784
	Female	17 (25.3)	12 (24.5)	5 (27.8)	1.0 (ref)	
Blood group	O	22 (32.8)	19 (38.8)	3 (16.7)	1.0 (ref)	
	A	19 (28.4)	10 (20.4)	9 (50.0)	5.70 (1.25–25.92)	0.024
	B	22 (32.8)	17 (34.7)	5 (27.8)	1.86 (0.39–8.99)	0.439
	AB	4 (6.0)	03 (6.1)	1 (5.6)	2.11 (0.16–27.58)	0.569
Body mass index (kg/m^2)^	<18.5	18 (26.9)	14 (28.6)	4 (22.4)	1.00 (ref)	
	18.5–22.9	28 (41.8)	22 (44.9)	6 (33.3)	0.96 (0.23–4.0)	0.949
	23.0–24.9	06 (09.0)	02 (04.1)	4 (22.4)	7.0 (0.92–53.23)	0.060
	≥25	15 (22.4)	11 (22.4)	4 (22.4)	1.27 (0.26–6.27)	0.767
Pre-existing comorbidities	Diabetes mellitus (DM)	34 (50.7)	23 (46.9)	11 (61.1)	1.78 (0.59–5.34)	0.304
	Hypertension (HTN)	61 (91.0)	43 (87.8)	18 (100)	—	0.181^$^
	Chronic liver disease (CLD)	4 (6.0)	2 (4.1)	2 (11.1)	2.937 (0.38–22.60)	0.301
	Chronic obstructive airways disease (COAD)	8 (11.9)	6 (12.2)	2 (11.1)	0.896 (0.164–4.90)	0.899
	Vascular disease (CAD/PVD)	17 (25.6)	9 (18.4)	8 (44.4)	3.56 (1.10–11.55)	0.035
	Chronic allograft dysfunction	21 (31.3)	14 (28.6)	7 (38.9)	1.59 (0.51–4.94)	0.422
	Obstructive sleep apnoea (OSA)	5 (7.5)	3 (6.1)	2 (11.1)	1.92 (0.29–12.53)	0.497
Acquired comorbidities	Cytomegalovirus (CMV) activation	3 (4.5)	0 (0.0)	3 (16.7)	—	0.017^$^
	Anti-fungal treatment^#^	18 (29.9)	4 (12.2)	14 (77.8)	39.8 (8.70–178.3)	<0.001
	Blood culture, +ve^##^	5 (7.5)	0	5 (27.8)	—	0.001^$^
	Urine culture, +ve^##^	4 (6.0)	0	4 (22.2)	—	0.004^$^
Baseline immunosuppression	CNI (Tac/CyA)	66 (98.5)	48 (97.9)	18 (100)	—	1.00^$^
	MMF/MPA	66 (98.5)	48 (97.9)	18 (100)	—	1.00^$^
	Steroids	67 (100)	49 (100)	18 (100)	—	—
Type of organ	Living donor	65 (97)	48 (97.9)	17 (94.4)	0.35 (0.02–5.98)	0.467^$^
	Cadaver donor	2 (3.0)	1 (2.0)	1 (5.6)	2.82 (0.17–47.68)	0.472
	Asymptomatic	4 (6.0)	4 (8.2)	0 (0.0)	—	0.567^$^
	Fever	59 (88.1)	43 (87.8)	16 (88.9)	1.12 (0.20–6.11)	0.899
	Cough	47 (70.1)	36 (73.5)	11 (61.1)	0.57 (0.18–1.78)	0.330
	Sore throat	18 (26.9)	11 (22.4)	7 (38.9)	2.20 (0.69–7.02)	0.184
	Body aches	25 (37.3)	18 (36.7)	7 (38.9)	1.10 (0.36–3.33)	0.872
Symptoms	Breathing difficulty	26 (38.8)	14 (28.6)	12 (66.7)	5.00 (1.57–15.94)	<0.001
	Loss of smell	4 (6.0)	4 (8.2)	0 (0.0)	—	0.567^$^
	Distaste	9 (13.4)	9 (18.4)	0 (0.0)	—	0.099^$^
	Headache	2 (3.0)	1 (2.0)	1 (5.6)	2.82 (0.17–47.68)	0.472
	Loose motions	5 (7.5)	3 (6.1)	2 (11.1)	1.92 (0.29–12.53)	0.497
	Extremes weakness	8 (11.9)	5 (10.2)	3 (16.7)	1.76 (0.38–8.27)	0.474
	Altered sensorium	3 (4.5)	0 (0.0)	3 (16.7)	—	0.017^$^

^*∗*^Odds ratio could not be computed due to zero count; ^$^Fisher's exact test; CAD/PVD, coronary artery disease/peripheral vascular disease; ^#^anti-fungal treatment when infection documented by positive urine or blood culture or suspected radiologically during active COVID-19 disease hospitalization or used in terminally sicker patients as empirical therapy; and ^##^bacterial culture positivity (blood or urine).

**Table 2:** Laboratory investigations in kidney transplant recipients with COVID-19.

Laboratory investigation	Number (N)	Mean (SD)	Min–max
Oxygen saturation (SpO_2_; %)	67	93.36 (5.4)	75–99
Lymphocyte (×10^9^/L)	64	12.68 (7.38)	1.0–45.70
Haemoglobin (gm%; Hb)	67	11.14 (2.05)	6.7–15.25
Total leucocyte count (TLC; cells/mm^3^)	67	9999 (4032)	4367–21433
Platelet count (×10^9^/L)	67	203.5 (76.5)	37.3–391
Blood urea (mg/dL)	67	77.27 (38.3)	21.06–178.0
Serum creatinine (mg/dL)	66	2.04 (1.31)	0.78–6.47
Aspartate aminotransferase (AST) (IU/L)	61	30.32 (11.62)	11.5–72.0
Alanine aminotransferase (ALT; IU/L)	62	33.86 (18.7)	8.0–93.5
Serum albumin (gm/dL)	63	3.55 (0.64)	1.8–4.7
Glycosylated haemoglobin (HBA1c; %)	31	7.08 (1.58)	5.0–11.9

**Table 3:** Management and treatment administered in kidney transplant recipients with COVID-19.

Parameters	Number	Percentage
Treatment parameters		
Hospitalization	41	61.2
Domiciliary	26	38.8
Room air management	33	49.3
Oxygen with mask	16	22.9
Noninvasive ventilator	5	7.5
Ventilator	14	20.9
Steroid	67	100
Azithromycin	29	43.3
HCQS	5	7.5
Ivermectin	39	58.2
Doxycycline	37	55.2
Tocilizumab	7	10.4
Remdesivir	23	34.3
Convalescent plasma	22	32.8

Thromboprophylaxis		
Anti-platelet	2	3
LMWH	31	45.3
OAC	18	26.9

Anti-nucleoside drugs		
Continued	10	14.9
Dose reduced	3	4.5
Drug stopped	51	76.1
Not taking	3	4.5

CNI drugs (tacrolimus or cyclosporine)		
CNI continued	48	71.6
CNI dose reduced	1	1.5
CNI stopped	18	26.9

Need for dialysis support (CRRT/SLEDD)		
AKI patients needing dialysis^*∗*^	13	20.6

Computerized tomographic scanning with CT score (*N* = 30)		
CT score <10	09	30
CT score 11–14	06	20
CT score ≥15	15	50
ICU requirement	19	28.4
Antibiotics used	41	61.2
Anti-fungal used	18	26.9

HCQS, hydroxy chloroquine sulphate, LMWH, low molecular weight heparin, OAC, oral anti-coagulants, and CNI, calcineurin inhibitors. ^*∗*^Patients with advanced graft dysfunction planned/initiated on HD during pandemic before PCR positivity were excluded.

**Table 4:** Association between mortality and investigations of kidney transplant recipients with COVID-19.

Parameter	Mean (SD)		Mean difference (95% CI)	*P*-value^*∗*^
	Survivors (*n* = 49)	Nonsurvivors (*n* = 18)		
Age (years)	49.18 (13.52)	57.22 (9.56)	8.03 (1.10–14.98)	0.024
Height (meter)	1.666 (0.08)	1.672 (0.08)	0.005 (−0.040 to 0.050)	0.814
Weight (kg)	69.18 (14.4)	73.5 (16.5)	4.32 (−3.93 to 12.56)	0.3
BMI (kg/m^2^)	20.8 (4.23)	21.7 (4.17)	0.94 (−1.38 to 3.26)	0.42
Transplant duration (weeks)	194 (117–370); *n* = 49	340 (262–448); *n* = 18	146 (25.0 to 227.5)	0.016
Haemoglobin (gm %; Hb)	11.46 (2.02)	10.27 (1.97)	−1.19 (−2.29 to −0.085)	0.035
Total leucocyte count (cells/mm^3^)	8820 (2717)	13211 (5228)	4391 (2437 to 6345)	<0.001
Platelet count (x10 (9)/L)	216.8 (75.18)	167.0 (69.53)	−49.85 (−90.44 to −9.26)	0.017
Serum creatinine (mg/dL)	1.84 (1.18)	2.58 (1.50)	0.74 (0.035 to 1.44)	0.04
Blood urea (mg/dL)	66.61 (32.99)	106.75 (37.42)	40.14 (20.79 to 59.48)	<0.001
Serum albumin (gm/dL)	3.71 (0.56)	3.09 (0.63)	−0.62 (−0.95 to −0.29)	<0.001
Lymphocytes (%)	14.02 (7.59)	8.98 (5.40)	−5.04 (−9.05 to −1.03)	0.015
Presentation SpO_2_ (%)	95.7 (2.78)	86.94 (5.77)	−8.77 (−10.86 to −6.68)	<0.001
AST (IU/L)	30.63 (11.53)	29.50 (12.15)	−1.13 (−7.81 to 5.56)	0.737
ALT (IU/L)	35.28 (19.60)	29.83 (15.90)	−5.55 (−16.19 to 5.09)	0.301

Inflammatory markers and other important parameters − median (interquartile range) difference (95% CI)				
IL-6 (pg/ml)	16.40 (4.10–79.10); *n* = 29	195.35 (64.7–891.4); *n* = 14	178.95 (50.3 to 692.2)	<0.001
Procalcitonin (ng/ml)	0.14 (0.05–0.48); *n* = 29	0.27 (0.11–2.84); *n* = 15	0.13 (−0.06 to 2.31)	0.101
D-dimer (ng FEU/ml)	590 (300–1350); *n* = 33	1402 (712–5563); *n* = 16	812 (27 to 4152)	0.002
CRP (mg/L)	34.45 (9.55–108.22); *n* = 28	149.6 (59.6–174.3); *n* = 14	115.11 (10.0 to153.6)	0.002
Ferritin (ng/ml)	522 (205–1438); *n* = 30	856 (715–1243); *n* = 16	333.65 (−197.0 to 745.5)	0.115
LDH (IU/L)	317 (257–413); *n* = 23	417 (291–806); *n* = 11	100 (−54.0 to 489.0)	0.091
Need of dialysis, *n* (%)	3 (6.12)	10 (55.6)	53.67 (10.71–269.04)	<0.001^$^
CT score ≥15^&^_,_*n* (%)	6 (31.6)	9 (82.8)	7.17 (2.04–25.22)	0.008^$^
Remdesivir	9 (18.4)	14 (77.8)	15.56 (4.13–58.57)	<0.001^$^
Tocilizumab	1 (2)	6 (33.3)	24.0 (2.63–218.67)	0.005^$^
Convalescent plasma	7 (14.3)	15 (83.3)	30.0 (6,86–131.19)	<0.001^$^
Ventilator need	0 (0)	14 (77.8)	—	<0.001^#^
Acute kidney injury (AKI), *n* (%)	8 (17.78)	16 (88.9)	37.0 (7.06–194.0)	<0.001^$^
ICU stay, *n* (%)	4 (8.2)	15 (83.3)	56.3 (11.3–280.6)	<0.001^$^

SpO_2_, oxygen Saturation, Hb, haemoglobin, TLC, total leucocyte count, IL-6, interleukin-6, LDH, lactate dehydrogenase, CRP, C-reactive protein. ^*∗*^Average of all available values.^&^CT score was available for 30, 19 survivor and 11 nonsurvivor. ^$^Odds ratio (95% confidence interval).

## Data Availability

All the data are well verified and authentic and available on request; please contact Dr. Sanjiv Jasuja at sanjivjasuja@yahoo.com.
